# EFFECTS OF ESSENTIAL CAREGIVER POLICIES ON COVID-19 AND NON-COVID-19 DEATHS IN NURSING HOMES

**DOI:** 10.1093/geroni/igad104.0121

**Published:** 2023-12-21

**Authors:** R Tamara Konetzka, Nadia Ghazali, Mingyu Qi

**Affiliations:** University of Chicago, Chicago, Illinois, United States; The University of Chicago, Chicago, Illinois, United States; The University of Chicago, Chicago, Illinois, United States

## Abstract

Balancing the safety of nursing home residents with their need for social interaction has emerged as a key challenge from the COVID-19 pandemic. Strict bans on nursing home visitation eventually led to growing concern that physical isolation may have unintended harms on nursing home residents. To address this concern, at staggered times between June 2020 and January 2021, 17 states implemented Essential Caregiver policies allowing nursing home visitation by designated family members or friends under controlled circumstances. Using the Nursing Home COVID-19 Public File and other relevant data, we analyze the effects of Essential Caregiver policies on COVID-19 and non-COVID-19 deaths for nursing home residents. To estimate plausibly causal effects, we employ recent innovations in difference-in-differences and event-study estimation. We find that on average, Essential Caregiver policies prevent 0.44 non-COVID-19 deaths, 0.42 COVID-19 deaths, and 0.86 total deaths per 1,000 residents in a two-week period, representing 8 percent, 17 percent, and 11 percent of the sample mean for the three outcomes, respectively. The effects are mainly driven by state policies that are mandatory or implemented without restrictions and are larger for high-quality facilities and highly staffed facilities. Our results suggest that nursing home residents derive substantial benefit from regular social interaction with family members and friends and from the additional care they provide. Our findings support the use and expansion of Essential Caregiver policies to balance resident safety and the need for social interaction for future infectious disease outbreaks.

